# Centrosomes and cilia in neurodegeneration: main actors or mere spectators?

**DOI:** 10.1098/rsob.240317

**Published:** 2025-05-21

**Authors:** Ramona Lattao

**Affiliations:** ^1^ Institute of Biochemistry and Cell Biology (IBBC), National Research Council (CNR), Monterotondo (Rome) 00015, Italy

**Keywords:** centrosome, neurodegeneration, cilia, neuron

## Abstract

Centrosomes are intracellular organelles traditionally recognized as the primary microtubule (MT) organizing centres (MTOCs) in the cell, playing a crucial role in organizing the cytoskeleton and forming the MT-based spindle during cell division. However, it is now well established that centrosomes also function as central hubs for a wide range of signalling pathways. In non-dividing cells, they give rise to the primary cilium, a surface antenna that serves as a key structure for signalling. Neurons are highly specialized cells with a distinctive morphology, and most neurons have cilia. During brain development, cilia regulate the self-renewal of neural progenitors, as well as the differentiation, migration and synapse formation of newly generated neurons. As a consequence, defects in cilia result in various neurodevelopmental disorders. The role of centrosomes and cilia in neurodegeneration, or the progressive loss of neurons, is less understood. Centrosomes take part in several cellular processes that are often disrupted in neurodegenerative diseases (NDDs), and many proteins associated with these conditions have been found at centrosomes or cilia suggesting a link between these organelles and the underlying mechanisms that contribute to neuronal decline. Unravelling if and how centrosome dysfunction contributes to neurodegeneration could significantly deepen our understanding of the underlying biology of these disorders. Such insights may pave the way for new therapeutic approaches to address these debilitating conditions.

**Figure:**
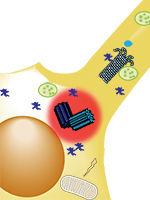

## 1. The players: centrosomes and cilia

Centrosomes are intracellular organelles consisting of two barrel-shaped centrioles surrounded by an amorphous and dynamic pericentriolar material (PCM) [1]. Historically, the centrosome has been defined as the main microtubule (MT) organizing centre (MTOC) of the cell, responsible for the organization of the cell cytoskeleton and the MT-based spindle during cell division. However, it is now clear that it is also an interaction hub for many cellular pathways.

The PCM hosts approximately 100 proteins, and its composition varies depending on the cell type and the stage of the cell cycle. Centrosomal proteins serve various critical functions, including cell cycle regulation, DNA-damage response, MT nucleation and trafficking. Together with proteins, RNAs, ribosomes and endosomes have been localized at the centrosomes. RNAs were first found at centrosomes in the 1960s, and decades later, the localization of a specific mRNA encoding cyclin B to the centrosomes of early *Drosophila* embryos was shown [2]. Pericentrosomal ribosomes were initially visualized via electron microscopy [3] and then confirmed across various systems by multiple researchers [4]. Moving along MTs, endosomes are often seen in the pericentrosomal region [5,6] where they are implicated in centrosome duplication and cilia formation or ciliogenesis [7].

Centrosome numbers within the cell must be tightly controlled. An interphase cell has one single centrosome that duplicates in S phase together with DNA and, after cell division, each daughter cell inherits a single centrosome. Extra centrosomes can result from aberrant cell division, cell fusion or uncontrolled centrosomal duplication during S phase. In either case, supernumerary centrosomes lead to defective chromosome segregation [8]. On the same line, besides few exceptions, cells need centrosomes to fulfil cell division and lack of centrosomes also has negative outcomes [9]. Indeed, centrosome abnormalities are a common feature of cancer, although for decades it was not clear if it was a cause or a consequence [8].

When the cell exits the cell cycle, the older (or mother) centriole becomes the basal body of the primary cilium, an antenna-like structure that protrudes from the cell surface and receives signals from the extracellular milieu. To fulfil this role, the ciliary membrane and the cilium content are enriched in signalling molecules (Sonic Hedgehog, Wnt, etc.). In certain cell types, cilia can be motile to ease the flow of extracellular fluids (e.g. respiratory epithelia) or to propel the cell (e.g. sperm, motile unicellular organisms).

Cilia direct cell migration during development, and mutations in ciliary proteins cause severe developmental multisystem disorders called ciliopathies [10]. One of the most severe conditions is Meckel–Gruber syndrome (MKS), a lethal autosomal recessive disorder, showing developmental anomalies including neural tube defects, skeletal malformations, congenital heart defects, and liver and kidney problems [11].

Bardet–Biedl syndrome (BBS) is a group of early onset autosomal recessive non-motile ciliopathies manifesting retinal dystrophy, obesity, intellectual disability, polydactyly, genital anomalies and renal malformations. Many of the BBS-causing genes encode for BBSome components, a protein complex implicated in ciliary vesicle trafficking [12].

Dysfunction of motile cilia results in primary ciliary dyskinesia/Kartagener syndrome characterized by impaired mucociliary clearance resulting in recurrent infections of the upper respiratory tract and progressive destruction of the respiratory tissue, male infertility, *situs inversus totalis*, heterotaxy and congenital heart disease [13,14].

Joubert syndrome (JS) is another rare autosomal recessive disease characterized by malformation of the cerebellum, hypotonia, intellectual disability, oculomotor apraxia, retinal dystrophy, physical malformations, abnormalities in the respiratory system, liver and kidneys [15].

Kidney abnormalities and renal fibrosis are also characteristic of autosomal dominant polycystic kidney disease (ADPKD), autosomal recessive polycystic kidney disease (ARPKD), nephronophthisis (NPHP) and NPHP-related ciliopathies [16].

## 2. Neurons and neurodegenerative disorders

Neurons are specialized cells with a peculiar morphology consisting of a cell body (soma), a main elongated projection (the axon) and several shorter extensions (dendrites). Each of these cellular districts performs a separate action: dendrites receive inputs and send signals to the soma, where the information is processed and then transferred via the axon to the receiving cell or neuron.

Neurons form and become terminally differentiated early during development [17]. During differentiation, one of the neurites is specified as the axon (neuronal polarization) in a process that relies on centrosomes [18]. Neuronal migration to their destination in the brain is regulated by the coordinated actions of actin filaments and MTs, with centrosomes directing this movement [19]. Indeed, mutations in centrosomal proteins cause microcephaly and other neurodevelopmental disorders.

Almost all neurons harbour cilia [20] although with different ultrastructures and lengths, suggesting specialized but so far uncharacterized functions. Moreover, cilia structure is very dynamic, and their length can be modulated according to circadian rhythm and metabolism [21].

Cilia control brain development by regulating the self-renewal of neural progenitors, and the differentiation, migration, and synapse formation of newly generated neurons [21]. Therefore, ciliopathies such as JS, MKS and BBS manifest neurodevelopmental defects [22].

Neurodegenerative diseases (NDDs) affect millions globally and are characterized by the progressive loss of neuronal function [23]. Currently, there are no treatments to halt their progression, and no cures exist. Common NDDs include Alzheimer’s disease (AD), Parkinson’s disease (PD), amyotrophic lateral sclerosis (ALS) and other motor neuron diseases (MNDs). The cellular mechanisms driving these conditions are complex and multifactorial. Although various risk genes have been identified and cellular markers of neurodegeneration, such as protein aggregates, have been reported, the precise triggering cause is still unknown.

Various proteins implicated in NDDs have been found at centrosomes or cilia, highlighting a potential link between these organelles and disease mechanisms. For example, a defining hallmark of AD is the accumulation of neurotoxic amyloid-β (Aβ) aggregates. The Aβ peptide is produced through the proteolytic cleavage of amyloid precursor protein (APP), which localizes to the primary cilium [24]. Similarly, presenilin 1 and 2 are linked to the majority of early onset familial AD and are known to associate with centrosomes [25]. TAR DNA-binding protein 43 (TDP−43), a highly conserved RNA/DNA-binding protein that accumulates in ALS, frontotemporal dementia (FTD) and AD that has been recently shown to be enriched at the centrosome [26] and ALS2, another protein linked to ALS, primary lateral sclerosis and ascending hereditary spastic paralysis, also associates with centrosomes [27]. Finally, mutant LRRK2, a common cause of PD, leads to deficits in centrosomal positioning and cohesion, adversely affecting neurite outgrowth, cell polarization and migration [28].

Despite significant progress, many questions still surround the role of centrosomes and cilia in these conditions. One of the primary challenges is the difficulty in distinguishing between developmental defects and neurophysiological abnormalities.

## 3. Centrosomes and cilia in neurodegenerative diseases

**Figure 1.**
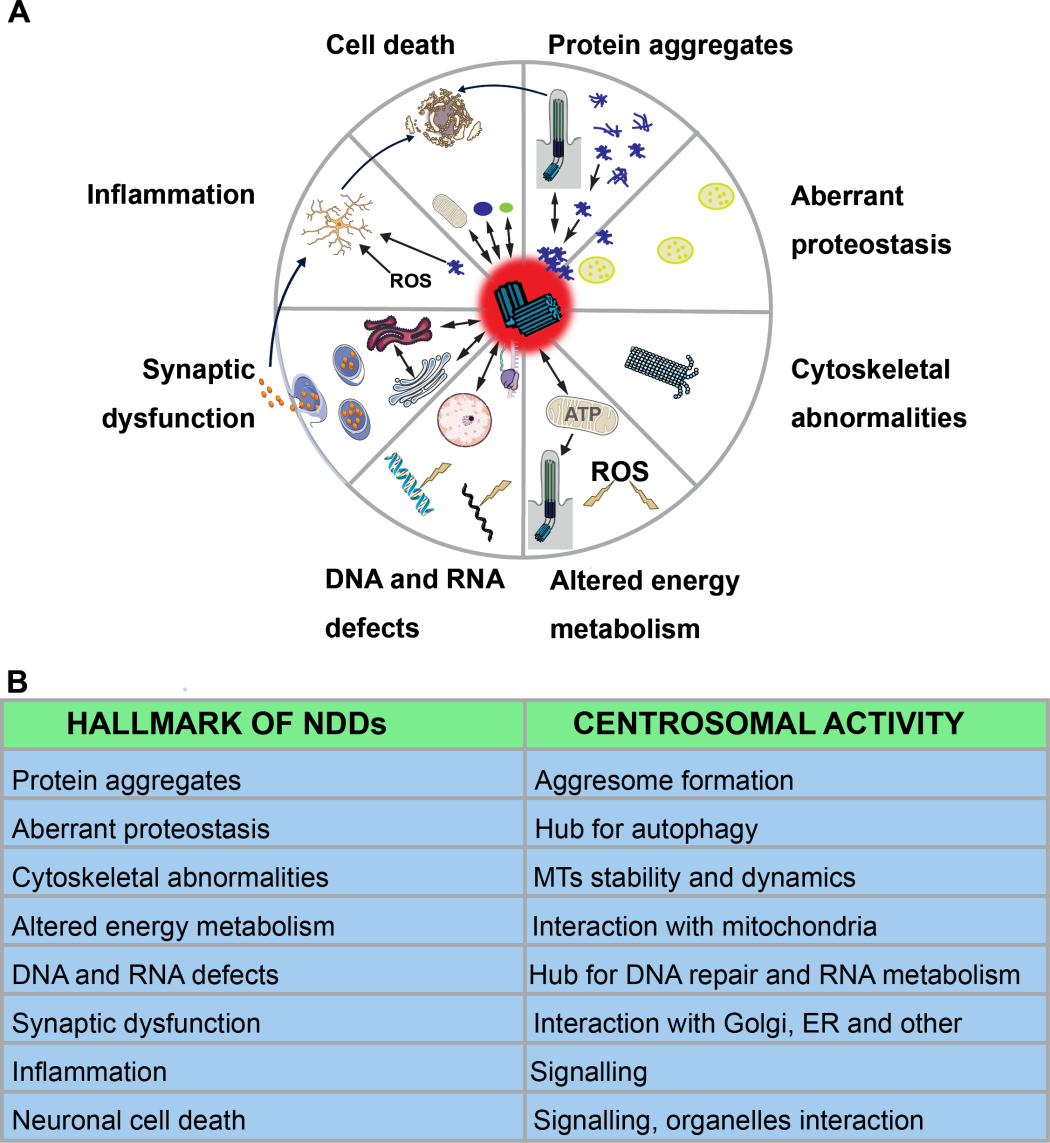
Eight hallmarks of neurodevelopmental disorders (NDDs) have been described [23]. (A) This schematic illustrates the involvement of centrosomes or cilia in each of the eight NDD hallmarks. Image elements were either created by the author or sourced from NIH BIOART (https://bioart.niaid.nih.gov/), Bioicons (https://bioicons.com/) or Servier Medical Art (https://smart.servier.com/). From NIH BIOART: Astrocyte (no. 40), Translation (no. 523), Cell Death (no. 71), Mitochondria (no. 352). From Bioicons (CC-BY 3.0 license)—Servier Medical Art (CC BY 4.0): Nucleus, Microtubule (modified), Golgi-2D-2, Rough Endoplasmic Reticulum-2, Exocytosis (modified). (B) The table summarizes the central role of the centrosome in each of these processes.

Currently, a set of interconnected hallmarks define NDDs: protein aggregation, aberrant protein homeostasis (proteostasis), cytoskeletal abnormalities, altered energy metabolism, DNA and RNA defects, synaptic dysfunction, inflammation and neuronal cell death [23]. It is still unclear whether defects in diverse processes converge on a single disrupted pathway or whether multiple independent pathways lead to the common outcome of neuronal degeneration. Notably, centrosomes play a role in all these affected processes (figure 1), and multiple studies have shown the co-occurrence of centrosomal and ciliary alterations in neurodegenerative conditions, suggesting a potential link between these organelles and the observed neuronal and glial cell phenotypes.

Protein aggregation is a hallmark of various NDDs, involving different proteins and affecting distinct brain regions. It is thought to contribute to both gain- and loss-of-function effects, although the precise toxic mechanisms are unclear [23]. Inside the cell, damaged proteins or organelles are degraded via the ubiquitin–proteasome system or the autophagy–lysosome pathway, respectively. In some NDDs, ubiquitinated proteins and dysfunctional lysosomes accumulate leading to cell death [23]. In cultured cells, potentially toxic misfolded proteins are directed to a pericentrosomal structure known as the aggresome, which eases their clearance via autophagy [29]. Aggresome formation is a complex, multi-step process that involves the recognition of misfolded and aggregated proteins, their coupling to the dynein motor complex, and the retrograde transport along MTs to the centrosome [29]. Aggresomes are in close relationship with Lewy bodies—intracytoplasmic aggregates seen in PD and dementia with Lewy bodies (DLB) neurons—that also incorporate the centrosomal proteins γ-tubulin and pericentrin [30]. An aggresome-like behaviour has also been described for mutated forms of fused in sarcoma (FUS), an RNA-binding protein (RBP) associated with ALS and other NDDs [31,32]. Evidence suggests that centrosomes take part in the cellular response to these aggregates. For example, when cells are treated with the proteasome inhibitor lactacystin, ubiquitinated proteins and parkin (an E3 ubiquitin ligase associated with PD) accumulate at the centrosome via a parkin–γ-tubulin interaction in a MT-dependent manner [33]. Given the centrosome’s role as a hub for autophagy, parkin may ubiquitinate substrates and direct their aggregation at the centrosome for efficient degradation.

In turn, aggregates affect the assembly of cilia resulting in impaired signalling and cell death [24], and recent work highlighted a connection between cilia movement and Aβ. APP can be cleaved at two different sites and originate two Aβ fragments of 40 (Aβ1−40) or 42 (Aβ1−42) amino acids. Aβ1−42 is more fibrillogenic than Aβ1−40 and its concentration is higher in AD brains. Aβ is transported into the cerebrospinal fluid (CSF) driven by ependymal ciliated cells. *In vitro*, Aβ1−42 affected the circadian rhythm of ciliary beating resulting in a decreased medium flow and enhanced neurotoxicity [34].

Mature neurons are characterized by long, uniformly oriented MTs in their axons, which serve as tracks for intracellular trafficking, supporting both axonal transport and synaptic function. These processes are particularly sensitive to changes in MT dynamics, and MTs destabilization has been linked to NDDs, including ALS [35,36], PD [37] and AD [38].

As neurons mature, centrosomes lose their role as MTOCs, and MTs are instead nucleated from Golgi outposts, cortical γ-tubulin complexes or pre-existing MTs [19,39]. Although centrosomes are not directly involved in MTs nucleation in mature neurons, they may still influence their stability and dynamics. This is supported by findings that in the presence of aggresomes, neurons are unable to nucleate MTs, organize their MTs network or form cilia [40]. Another evidence is NIMA-related kinase 1 (NEK1), a conserved kinase involved in cell cycle control, ciliogenesis and DNA damage response (DDR) that primarily localizes to the centrosome and at the primary cilium. Heterozygous variants in the gene account for 2–3% of both familial and sporadic ALS cases where it has been proposed to regulate MTs homeostasis and nuclear import [41].

Neurons are high-energy demanding cells. Energy is stored in adenosine triphosphate (ATP) molecules that capture chemical energy obtained from the breakdown of food molecules and releases it to fuel other cellular processes. ATP is generated by oxidative phosphorylation in mitochondria via the electron transport chain. Mitochondrial dysfunction is a prevalent feature of many NDDs, and it also affects ciliogenesis in neuronal cells [42,43]. Simultaneously, centrosomes regulate mitochondrial positioning and function. Given these observations, we can speculate that centrosome–mitochondria crosstalk is central to supporting both ciliogenesis and mitochondrial functionality. A key aspect of this relationship is the production of reactive oxygen species (ROS), which are by-products of cellular respiration capable of damaging DNA, RNA and proteins and have been linked to neurodegeneration [44]. High metabolic activity leads to increased ROS generation, making neurons particularly vulnerable to cell damage, and deficiency in the DNA repair mechanisms has been implicated in several neurodegenerative conditions. A representative example is TDP-43 that takes part in the detection of the DNA damage and in the assembly of DNA repair complexes by creating a scaffold for the recruitment of other DNA repair proteins [45]. Similarly, defects in RNA regulation including impaired RBP expression, cellular mis-localization and aggregation of RBPs are linked to neurodevelopmental dysfunctions and NDDs [46].

Synaptic activity is tightly regulated by a range of factors, including neurotransmitter release, calcium signalling, cytoskeletal dynamics, presynaptic vesicles transport, postsynaptic signalling, as well as mitochondrial function and energy supply [23]. Centrosomes engage in intricate interactions with various intracellular organelles, including the Golgi apparatus and endoplasmic reticulum (ER), which are vital for key cellular processes such as protein processing, vesicle trafficking and calcium homeostasis—all of which are crucial for synaptic function [47].

The presence of protein aggregates, damaged synapses or ROS may result in the activation of the microglia, the cells responsible for the brain defence system. Microglia closely interact with and activate astrocytes, which are critical for the maintenance of neuronal health and function. Indeed, neuroinflammation or the unresolved activation of the microglia (microgliosis) or the astroglia (astrogliosis) is a common feature of NDDs. Moreover, microglia rely on centrosomes for their activation and motility [48]. Dysregulation of centrosome function in microglia could therefore exacerbate neuroinflammatory responses.

All the above-described processes contribute to neuronal death, the common phenotype of NDDs.

## 4. Open questions

### 4.1. When and where does neurodegeneration begin?

The onset of neurodegeneration is still a major unanswered question, as most patients do not show noticeable symptoms until middle age, suggesting that defects may arise after neuronal differentiation. At cellular level, degeneration might start in the soma and spread to the neurites, or the reverse could occur. Although there are proven markers commonly used to assess neurodegenerative phenotypes, these often reflect terminal stages when cellular physiology is already compromised. Moreover, all the NDD hallmarks described are interconnected. As a result, pinpointing the first cause of degeneration is nearly impossible and likely varies across different conditions.

Cellular models provide an avenue to address this issue, enabling the parallel investigation of multiple pathways within a uniform genetic background. By gathering more detailed information, we may eventually move beyond the broad term ‘neurodegeneration’ and adopt a nomenclature that reflects distinct pathological pathways.

At the tissue level, an aspect to be investigated is whether the neurodegeneration is a cell-intrinsic or -extrinsic process originated from other cell types (e.g. immune system), and how the phenotype spread between cells.

To understand the role of centrosomes and cilia in NDDs, several aspects need to be explored. In this section, I highlight the most pressing areas of investigation, ranging from cellular mechanisms to their impact on tissue homeostasis. To enhance clarity, the questions have been grouped into four categories—biology of neuronal centrosomes, cell-autonomous mechanisms of neurodegeneration, tissue-level mechanisms of neurodegeneration and therapeutic approaches—while acknowledging that their implications often extend across these boundaries.

### 4.2. Biology of neuronal centrosomes

#### 4.2.1. How do centrosomes and cilia differ across neuron types, and how do these differences contribute to their vulnerability to neurodegeneration?

Cilia and centrosomes composition differs between cell types [21,49] and neurons are no exception. Neurons are highly specialized and diverse, with distinct types showing unique properties and functions. For example, rotenone-induced γ-tubulin aggregates are predominantly found in dopaminergic neurons, raising the question of whether these neurons are more sensitive to rotenone or predisposed to the formation of inclusion bodies [50]. Similarly, mouse models carrying the ALS-associated G93A SOD1 mutation show upregulated Wnt and TGFβ1 signalling pathways in astrocytes, while Notch signalling is decreased in motor neurons but elevated in astroglia [51]. Moreover, G93A SOD1 motor neurons in the spinal cord show a reduced number of cilia [51]. Cilia positioning within the tissue is another important aspect. Cilia receive external signals via G-protein-coupled receptors (GPCR) that rely upon the production of cyclic adenosine monophosphate (cAMP), cyclic guanosine monophosphate (cGMP) and other signalling cascades. Most ciliary GPCRs respond either to neuropeptides or neurotransmitters released within the synapse. Neuronal cilia are often found next to synapses where they can sense the chemical cues released in the surrounding and in mice axociliary synapse with serotoninergic neurons are involved chromatin remodelling [52].

Understanding the differences in centrosome and cilium function across various neuronal subtypes, along with their associated signalling pathways, could provide insight into why certain neurons are more vulnerable to degeneration in specific diseases. This knowledge may help uncover mechanisms underlying selective neuronal susceptibility and lead to targeted therapies.

#### 4.2.2. What is the role of centrosomes in the ageing brain, and how does this relate to neurodegeneration?

Ageing is a risk factor for NDDs, and alterations in centrosome function have been described in ageing cells [51,53]. Centrosomes undergo dynamic changes during the cell cycle and in response to cellular stress. The nature of these dynamics in post-mitotic neurons—particularly in the context of ageing—and their potential contributions to neurodegenerative processes require further investigation.

Additionally, environmental factors such as toxins, stress and lifestyle choices, including diet and exercise, can significantly influence cellular health. Exploring how these factors affect centrosome function in neurons and their potential role in neurodegeneration is a critical area for future research.

### 4.3. Cell-autonomous mechanisms of neurodegeneration

#### 4.3.1. How does centrosome or cilium dysfunction contribute to the onset and progression of NDDs?

Misfolded protein aggregation, a hallmark of many NDDs, has been linked to centrosome activity. Exploring the interactions between centrosomes and protein aggregates could yield insights into the cellular mechanisms driving neurodegeneration and potentially unveil new therapeutic paths.

Although centrosomes lose their MTOC activity in mature neurons, it is likely that they can still influence MT stability or dynamics, affecting intracellular transport and contribute to the spread of pathological proteins, worsening the disease.

Numerous studies show that centrosomes are involved in the cellular response to oxidative stress, and we have begun to elucidate the molecular mechanisms underlying this crosstalk [53], which may offer new insights into the processes underlying NDDs. Given their role as signalling hub, centrosomes or cilia dysfunction may activate signalling pathways that result in apoptosis (programmed cell death). Another aspect that needs to be investigated is the local protein synthesis at the centrosomes in the context of neuronal cells. Originally described decades ago, centrosomes are known to host all the components necessary for local protein synthesis, including RNA, ribosomes and RNA-interacting proteins [49] and recent studies have proven that local protein synthesis occurs at centrosomes [54,55]. This phenomenon is particularly significant in neurons due to their unique cellular morphology, and it is disrupted in NDDs [56]. For example, pericentriolar TDP-43 interacts with four centrosomal mRNAs together with 16 centrosomal proteins [26]. It is plausible that centrosomes support local protein synthesis within specific subcellular compartments, and any dysfunction at the centrosomal level could lead to an imbalance in protein homeostasis.

#### 4.3.2. How does centrosome dysfunction affect other cellular organelles, such as the Golgi apparatus, the endoplasmic reticulum and the nuclear envelope, in the context of neurodegeneration?

Disruptions in inter-organelles interactions may directly alt intracellular trafficking and adversely affect neuronal function and survival. Golgi apparatus is intimately connected with the centrosome and their interaction has a pivotal role in trafficking and cell polarity. During cell division, centrosomal components start a signalling pathway that leads Golgi fragmentation and coordinates Golgi dynamics with cell cycle progression [57]. Golgi fragmentation and apoptosis are often seen in NDDs where it is considered as the result of persistent Golgi stress [58,59].

Similarly, endoplasmic reticulum (ER) stress in NDDs results in the accumulation of misfolded proteins and alterations in calcium homeostasis [60]. Like Golgi, centrosomes control ER partitioning during cell division [61]. In *Caenorhabditis elegans*, centrosomes are surrounded by ER-derived membranes that form a structure called the centriculum (centrosome-associated membrane reticulum) next to the nuclear envelope (NE) [62]. It would be then interesting to search for centriculum-like structures in neurons and glial cells and to further characterize this interaction.

The NE is another structure closely associated with the centrosome throughout the cell cycle [63]. A genetic screen revealed that genes involved in NE integrity when inactivated, promoted tau aggregation, and when overexpressed, inhibited tau aggregation [64]. Moreover, NEK1-ALS neurons show defective transport of molecules between the nucleus and the cytoplasm [41]. It is thus conceivable that centrosomal dysfunction could trigger stress to these structures. However, the precise mechanisms and consequences remain to be elucidated.

### 4.4. Tissue-level mechanisms of neurodegeneration

#### 4.4.1. How do centrosome-related processes influence the blood–brain barrier in neurodegenerative diseases?

The blood–brain barrier (BBB) preserves the brain’s microenvironment, and its integrity is often compromised in NDDs [65]. Centrosomes support BBB integrity through their involvement in cell polarity and signalling. For instance, astrocytes, which are ciliated glial cells, are essential for the formation and maintenance of the BBB [66]. Investigating how centrosome dysfunction affects BBB function could uncover new dimensions of disease pathology and highlight potential therapeutic targets for intervention.

#### 4.4.2. What is the impact of centrosome-related changes on synaptic plasticity and memory formation?

Disruptions in centrosome function are known to impair axon and dendrite formation, which negatively affects synaptic connectivity and plasticity. Synaptic plasticity—the ability of synapses to strengthen or weaken over time—is crucial for learning and memory. By understanding how centrosome-related changes affect synaptic function, we can better understand the underlying mechanisms of memory impairment and find potential therapeutic targets for enhancing cognitive resilience.

#### 4.4.3. What is the role of centrosomes in neuronal injury responses and repair mechanisms?

Neurons have a limited ability for regeneration; however, they do activate various repair mechanisms following injury. Centrosomes may coordinate cellular responses to injury and help these repair processes. Understanding the functions of centrosomes in this context could be pivotal for developing therapeutic strategies aimed at enhancing neuronal repair in NDDs.

### 4.5. Therapeutic approaches

#### 4.5.1. Can centrosome abnormalities serve as biomarkers for early detection of neurodegenerative diseases?

Finding biomarkers for the early detection of NDDs is essential for effective management and intervention. Centrosome abnormalities may be detectable before clinical symptoms manifest, representing a promising avenue for early diagnosis. By figuring out the predictive value of centrosomal dysfunctions, we could enhance our ability to detect NDDs at an earlier stage, potentially improving patient outcomes through timely interventions.

#### 4.5.2. Can targeting centrosome-related pathways provide effective treatments for neurodegenerative diseases?

By focusing on centrosome-related pathways, we may uncover effective strategies to combat NDDs and improve patient outcomes. Given the vital role of centrosomes in supporting cellular homeostasis, investigating whether modulation of centrosome function through small molecules or genetic interventions can promote neuronal survival and resistance to stress could lead to innovative treatment options. Understanding the role of centrosomes in neuroinflammation may reveal new strategies for modulating inflammatory responses in NDDs. Enhancing neuronal resilience is another promising therapeutic approach. However, due to the diverse functions of centrosomes, it is essential to carefully consider potential side effects when developing such therapies.

## 5. Conclusions

The roles of centrosomes and cilia in neurons are still incompletely understood. Addressing these unresolved aspects will deepen our understanding of the molecular mechanisms underlying NDDs.

Exploring the evolutionary history of centrosomes and their neuronal functions may reveal why certain species are more susceptible to NDDs and uncover mechanisms that predispose to such conditions. By integrating evolutionary biology with neurodevelopmental research, we may find novel avenues for intervention and improve our ability to address these complex disorders.

## Data Availability

This article has no additional data.
